# Advances in the diagnosis of colorectal cancer: the application of molecular biomarkers and imaging techniques: a literature review

**DOI:** 10.1097/MS9.0000000000002830

**Published:** 2025-01-09

**Authors:** Alicia Su Huey Kwan, Olivier Uwishema, Sarah Mshaymesh, Karan Choudhary, Fatma K. Salem, Aman Singh Sengar, Raj Pravin Patel, Zeinab Kazan, Jack Wellington

**Affiliations:** a Department of Research and Education, Oli Health Magazine Organization, Research and Education, Kigali, Rwanda; b Department of Medicine for Older People, Southampton General Hospital, Southampton, United Kingdom; c Department of Natural Sciences, Faculty of Sciences, Haigazian University, Beirut, Lebanon; d Medical School, Department of General Medicine, MGM Medical College, Aurangabad, India; e Biochemistry Department, Faculty of Veterinary Medicine, South Valley University, Qena, Egypt; f Medical School, Department of General Medicine, Yerevan State Medical University after Mkhitar Heratsi, Yerevan, Armenia; g Department of General Surgery, Manohar Waman Desai General Hospital, Mumbai, India; h Faculty of Medical Sciences, Lebanese University, Beirut, Lebanon; i Department of Neurosurgery, Leeds Teaching Hospitals NHS Foundation Trust, Leeds, United Kingdom

**Keywords:** biomarkers, colorectal cancer, diagnosis, imaging, screening

## Abstract

**Background:**

Following neoplasms of the lung and breast, colorectal cancer (CRC) is the third most frequent malignancy globally. Screening for CRC at the age of 50 years is strongly encouraged for prompt earlier diagnosis owing to prognoses being greatly correlated with time of detection and cancer staging.

**Aim:**

This review aimed to elucidate the most recent advancements in the detection of CRC, with an emphasis on the latest innovations in diagnostic molecular biomarkers in conjunction with radiological imaging alongside stool-based tests for CRC screening.

**Methods:**

A comprehensive review of the literature was performed, focusing on specific terms in different electronic databases, including that of PubMed/MEDLINE. Keywords pertaining to “colorectal cancer,” “diagnosis,” “screening,” “imaging,” and “biomarkers,” among others, were employed in the search strategy. Articles screened and evaluated were deemed relevant to the study aim and were presented in the medium of the English language.

**Results:**

There have been several innovations in the diagnostics and identification of CRC. These generally comprise molecular biomarkers, currently being studied for suitability in disease detection. Examples of these include genetic, epigenetic, and protein biomarkers. Concurrently, recent developments in CRC diagnostics highlight the advancements made in radiological imaging that offer precise insights on tumor biology in addition to morphological information. Combining these with statistical methodologies will increase the sensitivity and specificity of CRC diagnostics. However, putting these strategies into reality is hampered by several issues.

**Conclusion:**

Progress in diagnostic technology alongside the identification of a few prognostic predictive molecular biomarkers suggested great promise for prompt detection and management of CRC. This clearly necessitates further efforts to learn more in this specific sector.

## Introduction

Colorectal cancer (CRC) accounts for nearly 10% of all cancer patients, making it the third most prevalent cancer and the second cancer type leading to deaths globally, according to the World Health Organization. An estimated number of CRC new cases surpassed 1.9 million, and CRC deaths surpassed 930 000 in 2020. An increase of 63% in the number of new cases/year and a jump to 73% in the number of deaths/year are predicted for the CRC burden by 2040^[1]^.

Significant geographical disparities in CRC incidence and mortality were detected, with Europe, Australia, and New Zealand at the top regarding the incidence rates and Eastern Europe regarding the mortality rates. It is worth mentioning that effective screening strategies in developed countries have contributed greatly to the falling CRC incidence, as early diagnosis, receiving proper treatment, and regular check-ups have enhanced CRC survival rates and life quality. Countries with low-risk populations, comprising Africa, Eastern Mediterranean Region, Latin America, and Asia, are predicted to witness the highest changes^[1,2]^.

Moreover, age is a significant factor for CRC, as older people aged 50 and above are the most affected individuals by CRC^[1]^. However, CRC incidence has recently witnessed a staggering rise since the mid-1990s in younger individuals, which has driven more attention to earlier screening^[2]^. Additionally, CRC development is thought to be hugely affected by environmental factors, such as lifestyle factors, including smoking, obesity, alcohol consumption, and intake of processed meats, fruits, and vegetables. Thus, leading healthy lifestyles, avoiding risk factors, and screening for early detection substantially reduce CRC incidence and negative outcomes^[1]^. Other factors, including CRC family history, familial adenomatous polyposis, Lynch syndrome, inflammatory bowel disease, gut microbiota dysbiosis, and chronic inflammation, are suggested to contribute to the development of CRC^[3]^.

## Molecular pathogenesis of CRC

**Figure 1.**
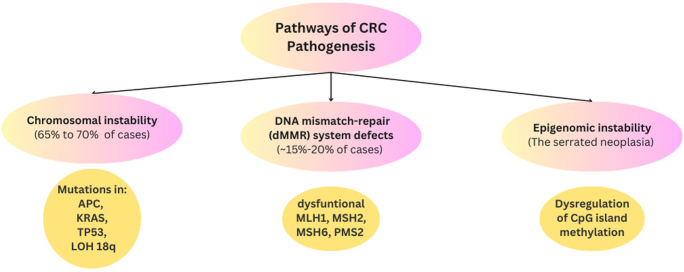
Pathways of CRC pathogenesis^[6]^.

As a multifaceted disease, CRC arises owing to a complex interplay of genetic, epigenetic, and environmental factors^[3,4]^. CRC pathogenesis mainly involves a minimum of three routes, which are chromosomal instability, microsatellite instability, and CpG island methylator phenotype pathways^[5]^, as mentioned in Fig. 1.

With a series of molecular changes, the typical pathogenesis of CRC involves the progression of the normal epithelium of the colon into invasive cancer. The Vogelstein and Fearon model originally proposed these progression sequences, which contributed enormously to understanding CRC molecular progression. It begins with the initiation of hyperproliferation of normal colonic epithelium, then the formation of different types of adenomas (early, intermediate, and late), followed by the formation of carcinoma and metastasis causing death. The authors suggested this pathogenesis sequence is stimulated by a series of mutations in a number of oncogenes and tumor suppressors, such as the adenomatous polyposis coli (APC) gene, in the initial step of transforming normal epithelium into early adenoma, followed by the accumulation of other gene mutations, such as *KRAS, TP53*, and loss of heterozygosity 18q, in the subsequent carcinogenic steps. The authors also highlighted the importance of the accumulation of these alterations rather than their order in CRC tumorigenesis^[6,7]^.

Multiple revisions were performed later to the original model, such as recognizing the potential of serrated polyps to transform into malignant tumors, not only tubular and tubulovillous adenomas as proposed earlier. While tubular adenomas are more commonly thought to be stimulated by genomic instability through suppressing the APC gene in addition to chromosome instability, premalignant serrated polyps are correlated with epigenetic alterations through the CpG island methylator phenotype, which represents aberrant hypermethylation of CpG dinucleotides underlining the heterogeneity of CRC and the involved genetic and epigenetic instability levels^[3]^.

## Importance of early detection in improving prognosis

Early detection of CRC by screening after 50 years of age is highly recommended, where disease prognosis is strongly associated with the subsequent timing of diagnosis and detected stage of disease. Less than 10% of individuals affected by CRC survive the later stages of disease progression (i.e., stage 4) compared to an estimated 90% of those who survive stage 1, according to a 5-year overall survival rate^[8]^. As CRC possesses an extensive period of incubation exceeding 10 years and a lack of clear symptomatology, most cases are diagnosed post-stage 3 (i.e., local lymphatic infiltration) or 4 (i.e., distant metastases). The overall 5-year survival rate drops to less than 10% in these situations. Moreover, only an approximate 25% of individuals with liver metastases – the most prevalent type of CRC metastasis – may benefit from appropriate surgical intervention. Hence, prompt identification is essential for increased patient survival and improved clinical outcomes, expanding subsequent therapeutic options available^[9]^.

## Current treatment effectiveness and diagnostic challenges

While radiation and chemotherapy are thought to be the mainstay of oncological management for patients with CRC, these interventions also greatly lower an individual’s quality of life, often associated with infection, dermatological sequelae, and/or nerve damage^[10]^. Now, stool-based testing and developments pertaining to radiological imaging are considered primary investigations for the screening of CRC in at-risk populations. Furthermore, owing to the advancements in “omics” as a means of analytical methodologies, an increasing number of molecular biomarkers have been recently observed to be of diagnostic benefit for CRC^[8]^.

Therefore, the purpose of this review is to gain a better understanding of the existing research pertaining to the diagnosis of CRC, in particular, early detection modalities. Molecular biomarkers provide a personalized approach to disease confirmation, aid prognostic evaluation, and provide insight into the probability of patient-centered treatment response. While improvements in radiological imaging techniques provide comprehensive, high-quality images, this review aims to elucidate whether said innovations contribute to reduced complications and augmented diagnostic accuracy.

## Molecular biomarkers in CRC

A plethora of prospective potential biomarkers for cancer diagnosis and prognosis have emerged alongside the breakthroughs in molecular pathology, genomics, and proteomics^[11]^. CRC biomarkers are usually acquired from liquid biopsies (genetic and epigenetic biomarkers) or tissue biopsies [including CTCs, cell-free deoxyribonucleic acid (cfDNA)/circulating tumor DNA, tumor-derived extracellular vesicles, autoantibodies, and tumor-educated platelets]^[12-18]^.

## Genetic biomarkers

### APC

About 85% of CRC cases are associated with the APC gene, and since APC is closely linked to the signaling system of Wnt/β-catenin, targeting Wnt/β-catenin signaling can be effective for CRC patients with APC mutations^[8]^. According to previous studies, the Wnt/β-catenin signaling pathway was blocked by retinoic acid-induced 2^[19]^, and controlled by a few other naturally occurring substances such as acetophenone and antibiotics^[20]^. Some other treatments and approaches were used for treating patients with APC mutations. Nevertheless, they were unable to completely halt APC-induced CRC in addition to the serious adverse effects of conventional medicine. Thus, there is a need to find new target genes for APC-driven CRCs^[8]^.

### Kirsten rat sarcoma virus

The mutation of the Kirsten rat sarcoma virus (KRAS) gene in cases of CRC is typically located at the 35th nucleotide of the 12th codon from Guanine to Thymine and is deemed to be closely associated with cancer invasion and lymphatic infiltration^[21,22]^. The KRAS gene plays a vital role in cell proliferation and differentiation processes, and it is necessary for epidermal growth factor receptor (EGFR) and RAS cellular signaling pathways. The KRAS gene is fundamental for the diagnosis and management of CRC since the mutation is observed in approximately 85% of cases^[22-24]^. According to recent research, patients can receive *in vitro*-expanded T cells to enable their immunity to recognize RAS-mutated specific antigens^[25]^.

### P53 gene

In colon cancer, the tumor protein gene (P53 gene) is mutated at a rate of 22–70%^[26]^. Moreover, the p53 gene is assumed to be associated with CRC distant metastasis and vascular invasion owing to the higher frequency of mutated p53 genes in distal colon tumors than in proximal ones^[27]^. According to recent research, a leucine-rich pentatricopeptide repeat-containing protein can be used as a therapeutic target for chemotherapy resistance induced by p53 mutations. Furthermore, in both *in vitro* and *in vivo* studies, the combination of 5-fluorouracil with gossypol-acetic acid, a particular inhibitor of leucine-rich pentatricopeptide repeat-containing protein, increased the drug’s efficacy^[28]^.

**Figure 2.**
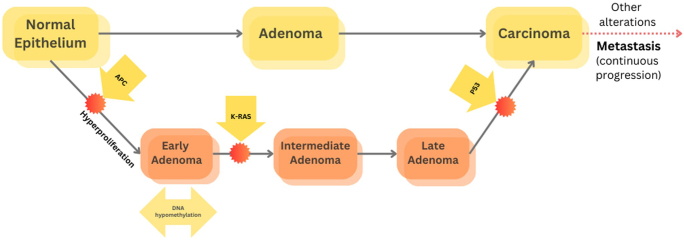
Molecular pathways in CRC development^[29]^.

Figure 2 shows the molecular pathways, mainly the mentioned genetic biomarkers, in CRC progression^[29]^.

## Epigenetic biomarkers

### Methylated DNA biomarkers

DNA methylation is one of many epigenetic modifications that have been exploited in the early detection and diagnosis of many diseases, including cancers^[30]^, whereby it is assumed to display a significant impact on CRC pathogenesis and disease progression^[31]^. Overall, cancer cells typically exhibit genome-wide hypomethylation alongside localized, specialized alterations (i.e., mostly hypermethylation) at regulatory regions such as promoters, often resulting in aberrant gene expression, functioning as cancer molecular biomarkers^[31]^.

A set of DNA methylation markers with a high level of accuracy and repeatability in a range of noninvasive and semi-invasive samples has been published throughout the prior decade. Researchers are currently working on creating a broad molecular biomarker panel to facilitate the diagnosis of CRC, possessing high sensitivity for differential diagnoses comprising CRC. Furthermore, it is thought that harnessing epigenetic modifications for oncotherapy in precision medicine will enhance curative abilities^[32]^.

### Specific markers

Although current research on employing DNA methylation for earlier detection of CRC remains fairly novel^[8]^, some studies have identified some potential methylated DNA biomarkers for prompt CRC diagnosis, including septin 9 gene (SEPT9), N-Myc downstream-regulated gene 4 (NDRG4), and bone morphogenetic protein 3 (BMP3). In addition, genetic methylation of wif-1, inositol monophosphatase 2 protein, and secreted frizzled-related protein 2 (SFRP2) exhibited high levels of specificity and sensitivity for CRC^[33,34]^. Moreover, it is reported that Syndecan-2 (SDC2) may play a vital role in the early diagnosis of CRC as its methylation expression was detected in higher levels in the earlier stages of CRC than in healthy tissue^[35-37]^.

SEPT9 is a guanosine triphosphate-binding protein and a member of the septin gene family whose role in the development of CRC is still under investigation. However, many reports have detected SEPT9 hypermethylation in CRC patients^[38,39]^. Interestingly, in CRC diagnosis, methylated SEPT9 (mSEPT9) exhibited higher sensitivity than other traditional markers such as fecal occult blood test, carbohydrate antigen 19-9, and carcinoembryonic antigen^[40-42]^, and the diagnostic sensitivity rose when m*SEPT9* was combined with any one of them, particularly in the early stages^[40,41,43]^. Although most studies reported relatively low mSTEP9 sensitivity between 8 and 40% for adenomas and polyps^[42,44]^, a recent research paper showed higher positive detection rates in adenomas and villous adenomas with significant dysplasia. The difference is thought to be attributed to the algorithm used in these studies^[45]^. Furthermore, mSEPT9 in plasma/serum was reported in several studies to have a sensitivity range of 47–87% and a specificity range of 89–98%^[42,46]^. This sensitivity remained low in early stages (I and II), and rose with higher stages, reaching 100% in stage IV^[44]^.

BMP3 is a member of a transforming growth factor-beta superfamily of cytokines, and it inhibits growth by affecting the suppressor of mothers against decapentaplegic family member 4 (SMAD4) gene transcription regulation. It was found that BMP3 downregulation might contribute to CRC early tumorigenesis^[47]^. NDRG4 is also involved in cell growth as well as differentiation. Like BMP3, NDRG4 was found to be downregulated in CRC^[48]^. Although using *5 mC* and *5hmC* as molecular markers is vital for the diagnosis of different cancer types, it has been hindered in the case of CRC by tiny sizes of samples and limited performance of the quantifying and analyzing methods used in addition to the loss of 5hmC in the advanced metastatic stages^[49,50]^. Nonetheless, it is noticed that the sensitivity of early-stage CRC can be improved by using 5hmC in multianalyte testing^[51]^.

Syndecan-2 (SDC2) is a member of the Syndecan family. It is also called fibroglycan and encodes a transmembrane type I heparan sulfate proteoglycan^[52]^. Frequent hypermethylation in the SDC2 promoter region has been found in the stool and blood samples of CRC patients^[53]^. DNA containing methylated *SDC2* extracted from stool holds great promise for the detection of noninvasive CRC with high specificity and sensitivity^[54,55]^.

The SFRP2 gene methylation in serum samples was found to be associated with a poor grade of differentiation, high tumor-node-metastasis stage coupled with +ve lymph node metastasis and deep cancer invasion in the wall tissue. Moreover, cases with positive SFRP2 hypermethylation in blood, stool samples, and tumor tissue exhibited lower overall survival compared to negative SFRP2 methylation cases^[56]^.

Interestingly, the combination of *SFRP2, BMP3, NDRG4*, and vimentin gene (*VIM*) methylation testing relatively increased the sensitivity and specificity for CRC. However, when it comes to the format of point of care, using biological samples for DNA methylation testing becomes challenging^[57,58]^.

## Micro ribonucleic acids

### Role of micro ribonucleic acids in CRC pathogenesis

Numerous studies have established a link between micro ribonucleic acids (miRNAs) and the development of CRC since microRNA (miRNA) expression is often lower in tumors than in healthy tissue^[59]^. There is growing evidence that miRNA plays an important role in CRC pathophysiology^[60,61]^, where miRNA controls the growth and dissemination of CRC via angiogenesis, cellular signaling pathways, and epithelial-mesenchymal transition (EMT)^[59]^. Several miRNA-mediated cellular signaling pathways, including the inactivation of APC and the overactivation of the Wnt/β-catenin pathways, thought to be the main contributors to CRC onset, are involved in the development and spread of CRC^[62,63]^.

### Diagnostic and prognostic potential of specific miRNAs

Abundant research has been conducted to examine miRNA expression patterns at either early or advanced stages of CRC^[64]^. For instance, elevated levels of miR-21, miR-29a, and miR-125b expression were associated with early CRC stages, high-grade intra-epithelial neoplasms, and tubular adenomas. In addition, they could be employed to differentiate advanced from non-advanced stages of CRC^[64]^. Moreover, some miRNAs were used to predict treatment response, which may facilitate the process of monitoring and predicting treatment efficacy, such as miR-126, whose elevated levels were predictive of poor response to bevacizumab (an anti-vascular endothelial growth factor monoclonal antibody), and decreased levels post-therapy were predictive of enhanced overall survival rates^[65]^. Here are some other examples of miRNAs and their prognostic/diagnostic value (Table 1)^[66-71]^:

**Table 1:** Examples of miRNAs and their prognostic and diagnostic value^[66-71]^.

miRNA level	Prognostic/diagnostic value	References
Elevated miR-141	Advanced stage IV CRC with a reported sensitivity of 66.7% and specificity of 84% in discriminating from stage I-II CRC	^[66]^
Elevated miR-221	Poor overall CRC survival and correlates with p53 expression	^[67]^
Elevated miR-29a	Early detection of liver metastasis	^[68]^
Elevated miR-203	Higher tumor-node-metastasis stage, lymph node, peritoneal, and distant metastasis with poor overall survival	^[69]^
miR-19a	Poor response and chemoresistance to the folinic acid, fluorouracil and oxaliplatin regimen	^[70]^
Elevated miR-204-5p	5-Fluorouracil sensitivity	^[71]^

## Protein biomarkers

### Circulating tumor cells

#### Recent advances in circulating tumor cell detection

As all cancer cells lack a common molecular biomarker, accurately detecting and isolating circulating tumor cells (CTCs) remains a profound challenge. Therefore, a plethora of methodologies have been developed to detect serum CTCs^[72]^, generally categorized into two main classifications: label-dependent and label-independent isolation techniques. While label-independent methods isolate CTCs without relying on present cell surface markers, they do so by successfully separating them from blood cells based on distinct biophysical features like size and deformability. Label-dependent methods use antibodies that target cell surface antigens in CTCs, which are distinguished by the presence of epithelial markers unlike normal blood cells^[73,74]^.

Epithelial cell adhesion molecule (EpCAM) is the most widely employed marker for CTCs in a variety of cancers as the source of most CTCs is epithelial cells^[74]^. Nevertheless, its usage as a CTC biomarker is limited as it cannot be used for cancers lacking or expressing EpCAM, like that of neurogenic cancers^[75]^, as well as CTCs with diminished EpCAM peri-EMT^[76]^. Furthermore, most EMT-related molecules are nuclear or cytoplasmic proteins, making them unfit for current membrane-based molecular tools. Consequently, single-cell CTC sequencing technologies are assumed to be advantageous in improving CTC isolation with a more thorough evaluation of the RNA-level EMT status of CTCs^[73]^.

#### Clinical significance in monitoring disease progression

Using CTCs through peripheral blood analysis has become widely recognized for tracking solid tumor progression. There are abundant studies confirming the diagnostic and prognostic value of CTC evaluation in different tumors, including CRC. However, differentiating CTCs in peripheral blood remains a real problem owing to their limited numbers in peripheral blood compared to other blood cells (i.e., nearly 1 CTC among 10 million WBCs/mL of blood in liquid biopsy)^[77]^. Furthermore, they are heterogeneous with various molecular markers^[78]^. Nevertheless, research is expanding, attempting to develop approaches to improve the overall survival rates of patients with elevated or negative shifts of CTC levels in addition to enhancing patient care^[73]^.

For instance, with the goal of enriching CTCs from whole blood, microfabricated trapping chambers are placed in microfluidic chip devices, and according to the variations between cancerous and healthy blood cells in size and shape, CTCs are isolated^[79]^. More confirming studies are still required, and the value of CTCs in CRC in screening and prognosis is still under investigation^[80]^. Nevertheless, a new CTC assay recorded an overall accuracy of 88% in all CRC stages, even in premalignant cases^[81]^.

Currently, CTC counts are predictive of progression-free survival and overall survival in different cancers including CRC^[73]^.

## Exosomal proteins

### Emerging role in noninvasive diagnosis and key proteins identified in recent studies

Most cancer patients currently receive treatment based on a precise diagnosis obtained through tissue biopsy and following genomic characterization^[82]^. However, tissue biopsies are prone to bias due to the heterogenic nature of cancers in addition to sampling difficulties in some cancers^[83,84]^. Exosomes are currently deemed a potential diagnostic marker for cancers due to their detectability in different fluids in the body, including blood, saliva, milk, and urine^[85]^. Abundant research shows that not only can exosomes be used as a powerful biomarker for CRC diagnosis, but also as a predictor of CRC development and metastasis, which is the primary reason for death in all cancer types. Furthermore, according to multiple studies, exosomes can also be used as a companion diagnostic tool to detect treatment response and efficacy^[86,87]^. Some exosomal biomarkers for CRC diagnosis were identified in recent studies such as elevated miR-1229^[88]^, circRHOBTB3^[89]^, CPNE3^[90]^, hsa_circ_0004771^[91]^, hsa_circ_0005100^[92]^, hsa_circ_0101802^[93]^, and has_circ_0010522^[94]^.

### Advances in imaging techniques

#### Conventional imaging technique

In addition to being one of the top three most common diagnostic findings in the population, CRC is the primary cause of cancer-related mortality, accounting for an estimated 900,000 fatalities annually^[95]^. Given the prolonged preclinical period of CRC, despite its high mortality rate, screening plays a critical role in lowering the number of cases^[95,96]^. The most prominent programs available for early diagnosis and screening have been colonoscopy and sigmoidoscopy^[95,96,97]^. Other screening techniques comprise colonic capsules, DNA testing, computed tomography (CT) colonography, and fecal occult blood/immunohistochemical testing^[95]^.

The influence of sigmoidoscopy has been demonstrated in trials to be efficacious in lowering prevalence; however, the effect is more noticeable on the left side of the colon than on the right^[96]^. Furthermore, sigmoidoscopy is more likely than colonoscopy to reveal any masses^[97]^. It has been demonstrated that colonoscopy is effective on both the left and right sides of the colon^[96]^. Out of all available screening techniques, colonoscopy is thought to be the most effective^[95]^.

Polypectomy, a procedure that removes non-cancerous growths during a colonoscopy, may be indicated to treat CRC in its early stages^[98]^. The cancer’s stage determines the respective mortality rate in CRC^[52]^. Not only are modalities like sigmoidoscopy and colonoscopy crucial for cancer staging, but they are also necessary for screening^[98]^. A retrospective study revealed that early cancer discovery led to gains in survival and a stage shift^[97]^.

Traditional screening methods, including fecal immunohistochemical, occult blood, and DNA testing, frequently exhibit limits in sensitivity and specificity. Additionally, other modalities, like invasive colonoscopy, have raised issues with ionizing radiation exposure during procedures and CT colonography^[97]^.

#### Advanced imaging techniques

Imaging techniques used in CRC cases have advanced from anatomical evaluation to detailed information pertaining to underlying tumor biology^[95]^. Innovation in radiological imaging has facilitated the evaluation of different phenotypes in generations of patients^[96]^. Also, based on the progression in imaging, predictive and prognostic findings are evaluated^[52,96,97,98]^. Conventional colonoscopy may only provide the outer morphology of masses without resolving the abnormal basal and subsurface microvasculature in CRC cases. Furthermore, cancer staging is performed by biopsy, which also shows a reduction in accuracy in cases of limited sampling^[98]^. The rate of false negatives increases in smaller polyps significantly^[97]^.

Several new modalities have been implemented to achieve high-quality, detailed images, one of which is chromoendoscopy, employing radio-opaque contrast dyes to study lesions, particularly those deemed to be flat^[96]^. High-definition colonoscopy integrated with a dedicated chromoscope has exhibited higher detection for adenomas, which are flat in nature, and for hyperplastic polyps^[97]^. High-definition modalities use 1080-line television with up to 1 million pixels and a wide-angle vision of 170°, which has been observed to be 30% more effective than conventional modalities^[98]^. High-definition colonoscopy also provides focus of up to 2 mm alongside magnification of approximately ×1.5; these specifications lead to mucosal enhancement^[52]^.

#### Radiological imaging and molecular imaging techniques

Powerful magnetic fields and radiofrequency pulses are used in magnetic resonance imaging (MRI) to produce images with good tissue contrast and spatial resolution^[99]^. MRI is a versatile technology that shows functional data along with structural and anatomical details, unlike CT, which is primarily a structural, morphological, and anatomical approach. Diffusion-weighted and dynamic contrast-enhanced MRI may be utilized to assess the biological and functional consequences of therapy^[100]^. Since 1959, the American Joint Committee on Cancer tumor-node-metastasis staging system has served as a unified structure for optimal care of patients with CRC. While traditional imaging has been the gold standard for cancer staging, positron electron tomography (PET) alongside conventional CT has been demonstrated to be more effective in assessing the main tumor, local/sentinel lymph nodes, and distant metastases. Tables 2 and 3 highlight the advantages and disadvantages of MRI in CRC staging^[101,102]^.

**Table 2:** Advantages of magnetic resonance imaging in the staging of colorectal carcinoma^[101,102]^.

Improved T-staging accuracy	In several studies it has been noted that PET has been outperforming CT imaging. This helps in selecting appropriate surgical approaches and better prognosis^[101]^
Enhanced N-staging sensitivity	When it comes to identifying regional lymph node metastases, PET/CT has a greater specificity than CT, which lowers the number of false-positive results^[101]^
Comprehensive evaluation	It allows a complete evaluation of the complete body which also helps to detect any metastasis that might otherwise go missed^[102]^
Noninvasive	Reduces the need for invasive procedures like biopsies while still providing detailed anatomical data^[102]^
Effective restaging tool	Enables the detection of the increased glucose metabolic rate that is characteristic of most of the malignant cells^[102]^

CT, computed tomography; PET, positron electron tomography.

**Table 3:** Cons of magnetic resonance imaging in the staging of colorectal carcinoma^[101,102]^.

Lower sensitivity in N-staging	Some studies have shown that it has a lower sensitivity compared to conventional CT methods, leading to a missed metastatic node^[101]^.
Physiological uptake interference	PET/CT image interpretation may be impaired due to differences in the uptake by organs like bowel/colon^[101]^.
Cost	High costs of these scans can lead to them being inaccessible in low-resource areas and population^[102]^.
Radiation exposure	Contains ionizing radiation, and a combined total of multiple scans can lead to a bad prognosis^[102]^.
Limited spatial resolution	May become a challenge when identifying lymph nodes near the primary tumor^[102]^.

CT, computed tomography; PET, positron electron tomography.

## Advanced optical imaging techniques

### Confocal laser endomicroscopy and fluorescence imaging

Confocal laser endoscopy (CLE) is a novel endoscopic instrument that facilitates microscopic resolution *in vivo* histology while conducting continuing endoscopy, as well as subsurface imaging of both normal and malignant human mucosa. The diagnostic and clinical management of patients scheduled for a screening or surveillance colonoscopy for CRC will undoubtedly be significantly impacted by this innovative approach. For example, CLE makes it possible to diagnose colonic neoplasms promptly, and the identification of neoplastic cells aids in focusing endoscopic intervention on the appropriate regions^[103]^. Some advantages of using CLE include being capable of producing 3D-sectioning by reducing out-of-focus light, providing rapid and noninvasive capture of high-resolution images, and creating 3D structures from images. However, it is comparatively expensive and has a restricted field of view, restricted to a few hundred microns^[99]^. A novel and emerging method named near-infrared (NIR) fluorescence imaging combines a NIR fluorescent agent with a specialized camera that may accentuate light in the NIR range. By enhancing the ability to distinguish between various structures, it seeks to achieve more accurate surgery with better oncological results and fewer issues. NIR fluorescent compounds may be employed topically or intravenously. An imaging system that uses fluorescence is implemented to image the indicated agent. This system comprises a specialized NIR stimulation light, collection optics and filters, and a camera for NIR fluorescence emission light in addition to a white light source and camera. In the operating room, a screen shows the NIR fluorescence output. It is preferable to have a concurrent visible light image that may be combined alongside the NIR fluorescence image^[104]^. NIR fluorescence is capable of producing images with excellent contrast and resolution and is observed to be flexible in selecting various fluorophores, having the capacity to view multiple fluorophores concurrently. However, it is not suitable for dense particles and must be completely stained to detect light. It also has relatively weaker fluorescence emission^[99]^.

### Integration of molecular biomarkers and imaging techniques

Early detection of CRC has been enhanced by utilizing multimodal diagnostic approaches, such as molecular biomarkers and radiological imaging techniques. The integration of high-throughput omics and statistical learning using advanced algorithms with multi-platform transcriptomics has created novel diagnostic signatures that may be employed in conjunction with radiological imaging techniques to increase the sensitivity and specificity of diagnosing CRC^[105]^. Methodologies comprising optical molecular imaging enable *in situ* lesion identification and improve disease detection through superior visual distinction between normal and neoplastic tissue. Moreover, this could be further integrated with the identification of specific molecular biomarkers identified from transcriptomic studies to develop a more comprehensive diagnostic tool^[106]^. The combination of new cell surface biomarkers, endoscopic techniques, and molecular imaging facilitates microscale visualization of the colon, which, in turn, may easily detect rare heterogeneous colorectal lesions that could be missed by conventional colonoscopy^[107]^. The development of noninvasive molecular biomarkers, particularly those found in stool, is highly accurate in detecting precancerous lesions and CRC. These methods of molecular detection, when combined with radiological imaging techniques, may augment screening program efficacy for CRC, potentially precipitating better clinical outcomes^[108]^.

CTCs and cfDNA are components of liquid biopsies that may be implemented in conjugation with conventional imaging strategies to detect early stages of CRC. The analyses of cfDNA and CTCs offer a noninvasive strategy to not only detect cancer-specific genetic alterations but also monitor tumor behavior during treatment. With this information, patients can receive diagnoses at an earlier stage, and clinicians subsequently may develop treatment plans that are patient-centered and personalized. Liquid biopsy is applicable in mutation locations among CRC patients. Unique genetic mutations that are not found in primary tumor tissue may be detected through cfDNA and CTC-DNA. This indicates that liquid biopsies can detect information concerning tumor heterogeneity and genetic profiling that may remain hidden or undiscovered by conventional biopsy or imaging modalities^[109]^. Analyses of cfDNA and CTC could provide valuable information about early diagnoses, the status of disease burden, and the risk of recurrence in earlier CRC stages. Thus, liquid biopsy in conjunction with radiological imaging techniques may enhance early diagnosis and holistic management of CRC^[80]^.

Recent research has demonstrated the value of integrated diagnostic techniques, which use genetic testing in conjunction with radiological imaging technology to facilitate accurate CRC diagnosis and therapeutic intervention. A personalized treatment plan that considers each patient’s unique molecular and biological landscape may be provided by combining liquid biopsies, preclinical avatars, and genomic, transcriptomic, and metabolomic analyses. By identifying traits unique to each patient, this method seeks to improve CRC diagnosis and therapy^[110]^.

The administration of chemicals tagged with fluorescence that bind preferentially to molecules expressed on or even in tumor cells has led to advancements in molecular imaging. It has been demonstrated that this method, which uses probes aimed at molecules such as c-Met, vascular endothelial growth factor, and EGFR, exhibits a high sensitivity for identifying CRCs. In clinical studies, the identification rate of colorectal adenomas using molecular imaging methodologies is greater than that of traditional observation using white light endoscopes^[111]^. Together, these findings show that there is a growing trend toward an integrated strategy based on molecular biomarkers to address CRC. This strategy combines sophisticated imaging methods with molecular data to assist in the management of this complex pathology more effectively.

## Emerging technologies

Application of modern modalities to comprehend the high variability and multifaceted mechanisms pertaining to oncogenetic heterogeneity, molecular diagnostics may provide extensive information on molecular profiling, aiding diagnosis and formulating specific therapy^[96,97]^.

Next-generation sequencing (NGS) applications in the world of oncogenesis have led to the understanding and diagnosis of specific genes responsible for cancer development alongside novel mutations, less frequent mutant genes, and epigenetic alterations^[112,113,114]^. The development of the “adenoma-carcinoma sequence” by Fearson and Vogelstein (1990) summarized the progression of non-cancerous polyp growth to a highly lethal invasive carcinoma attributed to the somatic mutations of APC, KRAS, BRAF, SMAD4, and TP53^[100,114]^. Among its application in the mechanism of oncogenesis, NGS is also applicable in liquid biopsies, particularly in cases of minimal residual disease^[112,114]^.

Based on genetic profiling, CRC is classified into different molecular subtypes, according to the “Consensus Molecular Subtypes Classification”^[95-97]^. Moreover, NGS facilitates the formulation of targeted therapy by identifying peculiar, mutated genes; one example of target therapy is EGFR inhibitors that are specific to cases without KRAS mutation^[52,98,112]^. Various clinical trials have based NGS solely, such as in genomic changes during therapy. By identifying inimitable mutations or alterations, we may develop more personalized therapies and provide precision to treatment^[113,114]^.

## Challenges and limitations in biomarkers and imaging technology

Radiological imaging in oncology has evolved for decades to lay the foundation for staging and targeted therapy^[95,96,97]^. Imaging modalities have expanded with the development of various contrast agents and novel molecular biomarkers^[52,98]^. Conventionally, the most widely employed radiological imaging technology is that of CT in cases of metastasis, and findings are assessed based on the Response Evaluation Criteria in Solid Tumors guidelines^[52,112]^. Various developments in advanced modalities have improved the detection of masses; however, these possess limitations, one of which is their availability to a vast population. Specificity and sensitivity are higher in advanced imaging modalities, but costs and access have become substantial barriers^[112,113]^.

Molecular biomarkers are vital for the detection of cancer; nevertheless, questions raised pertaining to their applicability and specificity to certain neoplasms, as in the case of the pediatric population, remain unanswered^[52,112]^. Despite the dawn of advancing radiological imaging, there are still no particular standards for each modality, which makes it challenging to manage each independent case^[97,98]^.

As the incidence of CRC among the young population of LMICs is increasing, implementing screening strategies for early detection has become mandatory. This raises attention to several factors that hinder the implementation of these techniques among these population, examples include weak infrastructure, diminished healthcare resources, barriers to proper accessibility to healthcare facilities, lack of standard diagnostic facilities, and shortage of healthcare practitioners, mainly gastroenterologists and pathologists. For that cooperation among medical leaders, advocacy groups, and educational systems is needed while building partnerships with HICs to build precision biomarker laboratories with cost-effective strategies to increase access to screening services^[115]^.

## Future directions

With the identification of predictive and prognostic molecular biomarkers for CRC diagnosis coupled with the progress made among molecular diagnostic technologies, it is evident that there is great potential for personalized medicine in CRC. Personalized medicine aims for maximally therapeutic effects with minimal adverse effects and individualizes treatment in a patient-centered manner. Examples comprise the use of patient-specific genetic material in CRC^[116]^. Molecular heterogeneity in CRC, including that of the microsatellite instability and hypermutation phenotype, is also significant in differences among patient treatment responses. High-throughput NGS technologies have contributed to a better understanding of tumor biological behavior and thus increased the precision of its prognostic and chemotherapeutic drug response analysis^[117]^. In addition, by detecting circulating tumor DNA, which indicates patients at higher risk of recurrence and those more likely to experience chemotherapeutic resistance, such recognition may aid in determining the most appropriate treatment course^[117]^.

Several additional important areas of emphasis pertaining to future developments in CRC diagnosis have been identified. Advancements in the development of novel targeted therapy and immunotherapeutic research are based on pre-existing literature regarding CRC as a heterogeneous disease with distinct tumor-specific characteristics affecting prognosis and clinical outcomes. Resultingly, these insights have shaped the scope of clinical trials for future treatments^[116]^. Hence, the search for more advanced diagnostic techniques that would allow earlier detection of CRC is ongoing. Furthermore, prognostic indicators that are superior to those in use now could be found. Tumor size, lymph node metastasis, and cancer cell properties are prognostic variables. These new indicators may help infer the most appropriate course of management, allowing further ease when assessing patient prognosis implicated in CRC^[117]^.

Research concerning CTCs may significantly aid CRC diagnosis and management. Future studies focusing on the identification and characterization of CTCs to improve prognosis and develop individualized treatment plans are warranted. Successful targeted therapies additionally require specialized analysis of the genetic and phenotypic profiles of CTCs, which are often different from those of the primary tumor^[118]^. We urgently necessitate novel emerging molecular biomarkers that aid the prognosis and diagnosis of CRCs. Further, studies examining the utilization of nanotechnology seem very promising in CRC diagnostics and therapeutics, where the development of new paradigms in nanotechnology for prompt CRC diagnosis and treatment remains of profound scientific interest. Studies aimed at improving the sensitivity and specificity of nanotechnology-based therapeutic applications might potentially improve patient outcomes and reduce mortality rates^[119]^. For these two important domains, molecular biomarker research must proceed and continue to thrive, encompassing molecular biomarkers generated from tumor tissue alongside those based on liquid biopsies. Proposed primary objectives for future study direction may be to validate and apply these molecular biomarkers in clinical settings for early identification, follow-up, and precision treatment of CRC^[120]^. Nonetheless, there are various important issues that must be resolved to ensure the successful implementation of these novel technologies in the health systems of low- to middle-income countries. Laboratories and training facilities for genetic testing and analysis are basic needs that require serious infrastructure development. Evaluating the cost-effectiveness of genetic technologies is also imperative so as to ensure access and context relevance in local settings. There needs to be education and training for healthcare providers, supportive regulatory frameworks, and public awareness campaigns promoting acceptance of the concept of genetic testing.

## Conclusion

A combination of its poor prognosis, morbidity, and mortality rates, CRC is renowned as one of the most fatal neoplasms globally. These issues are mostly caused by a failure to detect CRC in a timely manner. Prompt diagnosis is therefore an essential measure for reducing the mortality rate associated with CRC. However, because early indicators of CRC are not always evident and diagnosis is often influenced by other intestinal pathologies like that of colitis, CRC diagnostics remain challenging in modern medicine. We have reviewed and described several developments in the diagnosis and detection of CRC in this review, including the application of molecular biomarkers, such as protein, genetic, and epigenetic markers for detection, alongside highlighting the latest developments in radiological imaging modalities. To aid in early detection, this review also integrates imaging techniques with molecular biomarkers.

It is now possible to investigate potential clinical implications as diagnostic or therapeutic targets in CRC in greater detail thanks to an improved understanding of the regulatory processes governing cancer-specific epigenetic modifications. There is still a need for improvement in the diagnostic performance of said molecular biomarkers when it comes to prognosis, response to treatment prediction, or detection of early CRC stages. As these may lessen the reliance on conventional biopsy-based techniques, modern imaging technologies provide the foundation of most therapeutics established to support the detection and follow-up of all phases of treatment in all forms of cancers, including solid tumors. The ability of labeling methodologies to distinguish between cancerous and healthy cells is fundamental to the efficacy of imaging technologies. With the development of more sophisticated diagnostic biomarkers and imaging techniques utilizing these proteomic/genomic biomarkers, next-generation therapeutics will be available, providing far more effective detection and treatment alongside a reduced likelihood of disease relapse in the near future for most cancers.

## Data Availability

Not Applicable
